# Assessment of Mechanisms Involved in Antinociception Produced by the Alkaloid Caulerpine

**DOI:** 10.3390/molecules190914699

**Published:** 2014-09-16

**Authors:** Luiz Henrique Agra Cavalcante-Silva, Maria Alice Pimentel Falcão, Ana Carolina Santana Vieira, Max Denisson Maurício Viana, João Xavier de Araújo-Júnior, Jéssica Celestino Ferreira Sousa, Tania Maria Sarmento da Silva, José Maria Barbosa-Filho, François Noël, George Emmanuel C. de Miranda, Bárbara Viviana de Oliveira Santos, Magna Suzana Alexandre-Moreira

**Affiliations:** 1 Laboratory of Pharmacology and Immunity, Institute of Biological Sciences and Health, Federal University of Alagoas, Maceió 57020-720, Brazil; E-Mails: luiz0710@gmail.com (L.H.A.C.-S.); falcao.map@outlook.com (M.A.P.F.); carolinavieira2708@gmail.com (A.C.S.V.); viana.mdm@gmail.com (M.D.M.V.); 2 Laboratory of Medicinal Chemistry, Posgraduate Program in Pharmaceutical Sciences, Federal University of Alagoas, Maceió 57020-720, Brazil; E-Mail: jotaaraujo2004@gmail.com; 3 Postgraduate Program in Natural Products and Synthetic Bioactive, Federal University of Paraíba, João Pessoa 58051-900, Brazil; E-Mails: jessicacelestino26@hotmail.com (J.C.F.S.); jbarbosa@ltf.ufpb.br (J.M.B.-F.); 4 Molecular Sciences Department, Federal Rural University of Pernambuco, Recife 52171-900, Brazil; E-Mail: taniasarmento@dcm.ufrpe.br; 5 Department of Pharmaceutical Sciences, Federal University of Paraíba, João Pessoa 58051-900, Brazil; 6 Laboratory of Biochemical and Molecular Pharmacology, Institute of Biomedical Sciences, Federal University of Rio de Janeiro, Rio de Janeiro 21941-912, Brazil; E-Mail: fnoel@pharma.ufrj.br; 7 Laboratory of Marine Algae, Department of Systematics and Ecology, Federal University of Paraíba, João Pessoa 58051-900, Brazil; E-Mail: mirandag@dse.ufpb.br

**Keywords:** caulerpine, antinociception, adrenergic pathway, serotonergic pathway

## Abstract

In previous works we showed that oral administration of caulerpine, a bisindole alkaloid isolated from algae of the genus *Caulerpa*, produced antinociception when assessed in chemical and thermal models of nociception. In this study, we evaluated the possible mechanism of action of this alkaloid in mice, using the writhing test. The antinociceptive effect of caulerpine was not affected by intraperitoneal (i.p.) pretreatment of mice with naloxone, flumazenil, l-arginine or atropine, thus discounting the involvement of the opioid, GABAergic, l-arginine-nitric oxide and (muscarinic) cholinergic pathways, respectively. In contrast, i.p. pretreatment with yohimbine, an α_2_-adrenoceptor antagonist, or tropisetron, a 5-HT_3_ antagonist, significantly blocked caulerpine-induced antinociception. These results suggest that caulerpine exerts its antinociceptive effect in the writhing test via pathways involving α_2_-adrenoceptors and 5-HT_3_ receptors. In summary, this alkaloid could be of interest in the development of new dual-action analgesic drugs.

## 1. Introduction

Over the last decades an enormous number of marine natural products have been studied [1]. A previous study from our group has shown that extracts obtained from *Caulerpa* spp. cause significant antinociception in mice when assessed in both neurogenic and inflammatory nociception models [2,3,4]. We have also reported that one of the major constituents isolated from *Caulerpa* spp. extracts, the bisindole alkaloid caulerpine (5,12-dihydrocycloocta[1,2*b*;5,6*b*']diindole-6,13-dicarboxylic acid dimethyl ester), produces antinociception when assessed against chemical (acetic acid and formalin) and thermal stimuli in mice, without affecting the motor response of these animals. This alkaloid also prevents the inflammatory process induced by zymosan and capsaicin [2]. Furthermore, we demonstrated that caulerpine inhibits serotonin-induced contractions in the guinea pig ileum [5]. Although isolated mainly from green algae of the genus *Caulerpa* [6], we note that caulerpine has also been reported in other green (*i.e*., *Codium decorticatum* and *Halimeda incrassate*) [7,8] and red (*i.e*., *Chondria armata*) algae [9].

We previously described the antinociceptive and anti-inflammatory activities of caulerpine [2], but without evidence of its mechanism of action, which could involve different pathways reported to be relevant in this context, such as the opioid, serotoninergic and adrenergic pathways. The opioid system is an important system involved in pain control since opioid agonists activate intracellular signaling (*i.e*., suppression of adenylyl cyclase (AC) activity and a negative and positive influence on Ca^2+^ and K^+^ channels, respectively), leading to a reduction in the excitability of neurons and pain inhibition [10,11].

Another important system involved in descending control of pain is the noradrenergic pathway, mainly through the activation of α_2_-adrenoceptors, leading to inhibition of AC, suppression of Ca^2+^ currents and facilitation of K^+^ currents [12]. The involvement of muscarinic cholinergic receptors has also been reported as in the dorsal horn and through supraspinal mechanisms modulating nociception by the activation of the noradrenergic pathway of descending inhibition [13]. GABA displays a crucial and complex role in the modulation of the nociceptive process, especially in response to acute and chronic noxious stimulus [13].

The l-arginine-nitric oxide pathway is another system to be considered since it has been implicated in several experimental models used for pain evaluation. Nitric oxide (NO) plays a dual role in the nociceptive system and its pro- or antinociceptive effect depends of many features: (1) experimental model used; (2) dose of NO donors or precursor; and (3) administration route of NO donors [14]. Finally, the serotonin (5-HT) system displays a complex framework in pain regulation. The actions of 5-HT in the dorsal horn have been considered suppressive to the nociceptive process, mainly through activation of the 5-HT_3_ receptor [15]. On the other hand, activation of this receptor at the spinal cord may involve enkephalinergic dorsal horn neurons, which can explain part of the antinociception elicited by 5-HT_3_ stimulation [13,16]. In this study, we assessed the possible involvement of all these pathways in the antinociceptive action of caulerpine, mainly using pharmacological tools and the writhing test.

## 2. Results and Discussion

To evaluate the mechanism of action of the antinociceptive effect of caulerpine, we chose the writhing test, a model where nociception is induced by acetic acid [17]. After acetic acid injection, the mice show a response characterized by abdominal constriction, which is sometimes accompanied by twisting of the trunk followed by extension of the hind limbs [17]. This behavior results from the activation of acid-sensitive ion channels (ASICs) and transient receptor potential vanilloid 1 (TRPV1) localized in afferent primary fibers [18]. Furthermore, acetic acid injection induces a release of TNF-α, interleukin 1β and interleukin 8 by resident peritoneal macrophages and mast cells [19], as well as prostanoids and bradykinin [20,21]. It has also been reported that spinal MAP kinases (ERK, JNK and p38), PI3K, and microglia mediate acetic acid-induced writhing response in mice [22]. Using this model, we tested the capacity of classical antagonists of different pathways involved in nociception to block the antinociceptive effect of caulerpine. It is noteworthy that caulerpine caused a dose-dependent reduction of abdominal constrictions induced by acetic acid in the mice [2]; however in this study we choose a single dose (40 mg/kg) to carry out the experiments described below.

**Figure 1:**
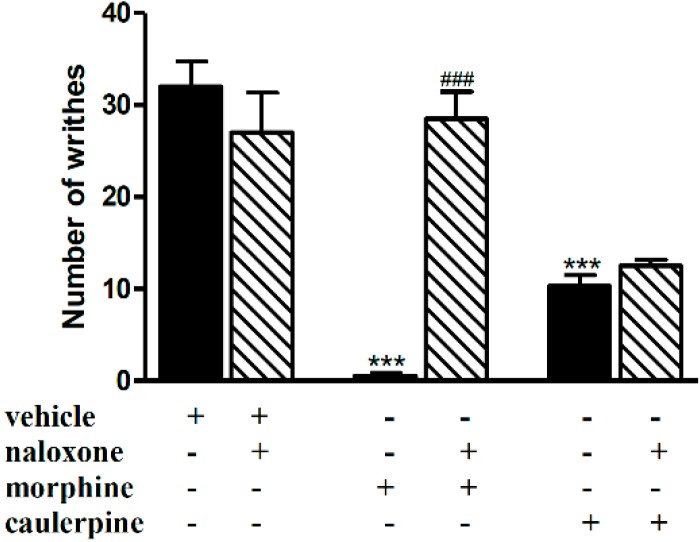
Effect of naloxone on the antinociceptive effect of caulerpine in the acetic acid model of nociception. Black columns: treatments alone; hatched columns: pretreatment and treatments. The results are expressed as means ± S.E.M. of six animals. (One-way ANOVA followed by Bonferroni’s test). *** *p* < 0.001 compared to vehicle; ^###^ *p* < 0.001 compared to morphine.

The systemic pretreatment of the animals with naloxone (1 mg/kg, i.p.), completely blocked the antinociceptive effect of morphine (5 mg/kg, i.p.) but not caulerpine (40 mg/kg, p.o.) when animals were challenged with acetic acid (Figure 1). The involvement of the opioid system in caulerpine-induced antinociception in mice was thus discarded, since the nonselective opioid receptor antagonist naloxone failed to affect the antinociceptive effect of this alkaloid. Unlike our findings other studies have reported an antinociceptive effect of indole alkaloids through the opioid system [23,24]. Despite belonging to the same secondary metabolite class, caulerpine could conceivably be metabolized to active compound(s) lacking opioid activity.

**Figure 2:**
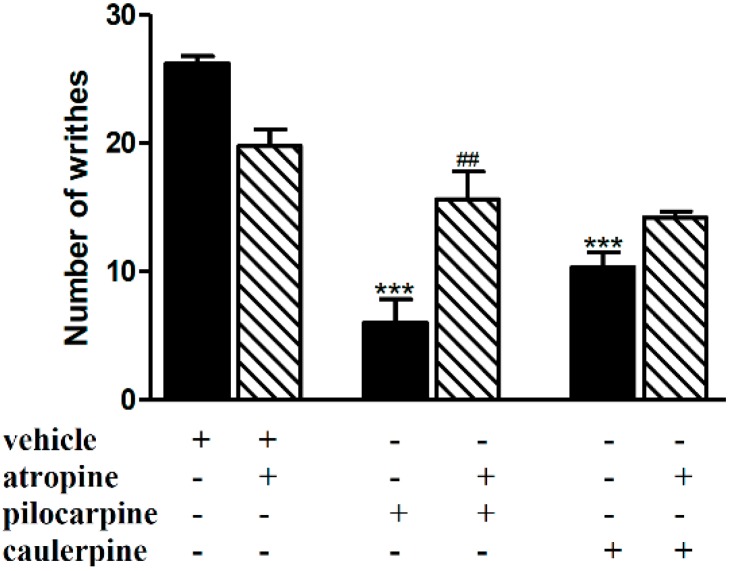
Effect of atropine on the antinociceptive effect of caulerpine in the acetic acid model of nociception. Black columns: treatments alone; hatched columns: pretreatment and treatments. The results are expressed as means ± S.E.M. of six animals. (One-way ANOVA followed by Bonferroni’s test). *** *p* < 0.001 compared to vehicle; ^##^ *p* < 0.01 compared to pilocarpine.

Similarly, three other pathways involved in the control of pain (the cholinergic, GABAergic and l-arginine-NO pathways) were also discarded as possible mechanisms of the antinociceptive effect of caulerpine, based on the following sets of data:

Pretreatment of mice with atropine (1 mg/kg, i.p.), the classical antagonist of muscarinic receptors, antagonized the antinociception caused by pilocarpine (3 mg/kg, i.p.) but not caulerpine (40 mg/kg, p.o.), in the acetic acid model of nociception (Figure 2).

**Figure 3:**
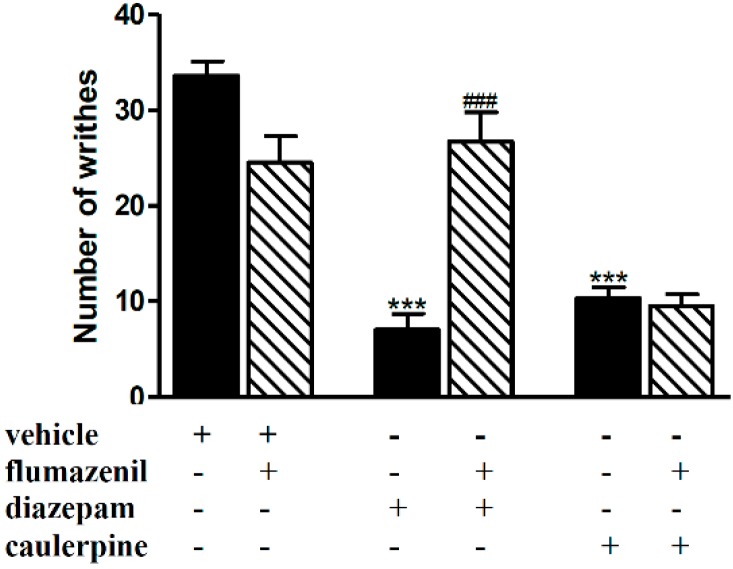
Effect of flumazenil on the antinociceptive effect of caulerpine in the acetic acid model of nociception. Black columns: treatments alone; hatched columns: pretreatment and treatments. The results are expressed as means ± S.E.M. of six animals. (One-way ANOVA followed by Bonferroni’s test). *** *p* < 0.001 compared to vehicle; ^###^ *p* < 0.001 compared to diazepam.

In the same way, the systemic treatment of the animals with flumazenil (2 mg/kg, i.p.) blocked the antinociceptive effect of diazepam (1.5 mg/kg, i.p.) but not caulerpine (40 mg/kg, p.o.) (Figure 3). In fact, this effect of caulerpine was not inhibited by the pretreatment with the classical antagonist of GABA_A_ receptors (flumazenil) which fully inhibits the antinociceptive effect of diazepam, used as positive control in paired experiments.

**Figure 4:**
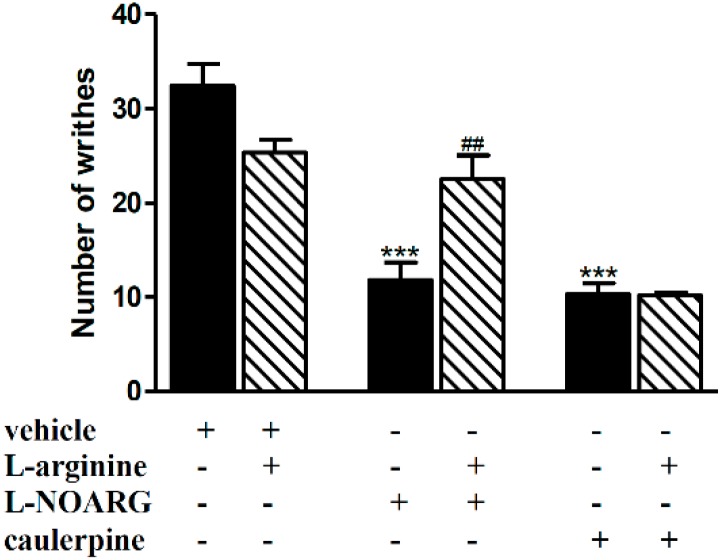
Effect of l-arginine on the antinociceptive effect of caulerpine in the acetic acid model of nociception. Black columns: treatments alone; hatched columns: pretreatment and treatments. The results are expressed as means ± S.E.M. of six animals. (One-way ANOVA followed by Bonferroni’s test). *** *p* < 0.001 compared to vehicle; ^##^ *p* < 0.001 compared to l-NOARG.

Figure 4 shows that l-arginine (40 mg/kg, i.p.) significantly blocked the antinociceptive effect of l-NOARG (75 mg/kg, i.p.) but not caulerpine (40 mg/kg, p.o.) after acetic acid challenge. Based on the literature [25] and our results, NO seems to exert an antinociceptive effect in the writhing test since the pretreatment with l-arginine, a NO precursor, inhibited the nociception induced by l-NOARG, a known nitric oxide synthase inhibitor. Our data also indicate that the antinociceptive effect of caulerpine does not involve the liberation of NO.

Contrary to the above results discarding the involvement of the cholinergic, GABAergic and l-arginine-NO pathways in the antinociceptive effect of caulerpine in the writhing test, the following sets of results indicate a contribution of the adrenergic and serotonergic systems.

**Figure 5:**
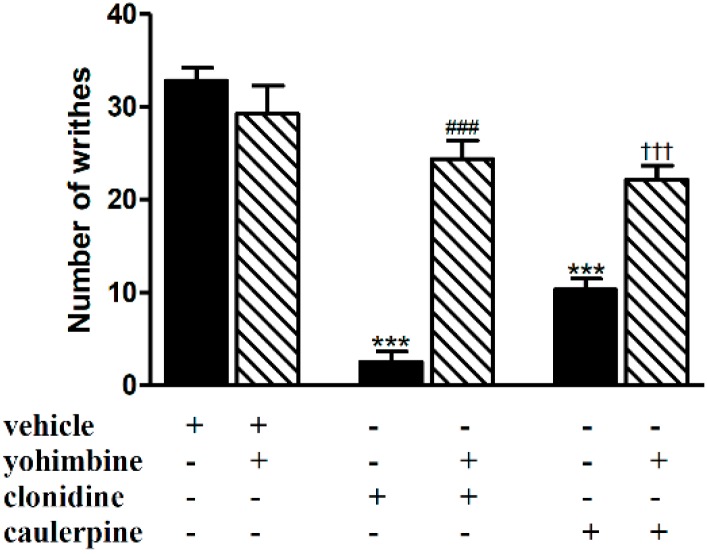
Effect of yohimbine on the antinociceptive effect of caulerpine in the acetic acid model of nociception. Black columns: treatments alone; hatched columns: pretreatment and treatments. The results are expressed as means ± S.E.M. of six animals. (One-way ANOVA followed by Bonferroni’s test). *** *p* < 0.001 compared to vehicle; ^###^ *p* < 0.001 compared to clonidine; ^†††^ *p* < 0.001 compared to caulerpine.

Figure 5 shows that pretreatment with the classical α_2_-adrenoceptor antagonist yohimbine (0.15 mg/kg, i.p.) largely antagonized the antinociception caused by clonidine (0.15 mg/kg, i.p.) and caulerpine (40 mg/kg, p.o.). Although caulerpine is an indole compound, like yohimbine, our result did not prove that caulerpine acts as a direct agonist of the α_2_-adrenoceptor. Indeed, we were able to test directly an effect of caulerpine on the α_2_-adrenoceptors, using a radioreceptor binding assay. At the highest concentration tested (3 µM, being limited by the low solubility of this alkaloid and the need to maintain a low final concentration of DMSO in the assay), caulerpine had no effect on the binding of 1 nM [^3^H]-RX821002 to our preparation of rat brain cortex, indicating that it does not bind to α_2_ adrenoceptors, at the highest concentration used (3 µM, data not shown).

Note that α_2_-adrenoceptor agonists, such as clonidine, also act through indirect pathways, such as the serotonergic system [26]. Since the acetic acid model of nociception is an *in vivo* experiment, caulerpine could be metabolized to an active compound(s), such as the corresponding acid or may be another active derivate of phase I metabolism, with α_2_-adrenergic activity.

**Figure 6:**
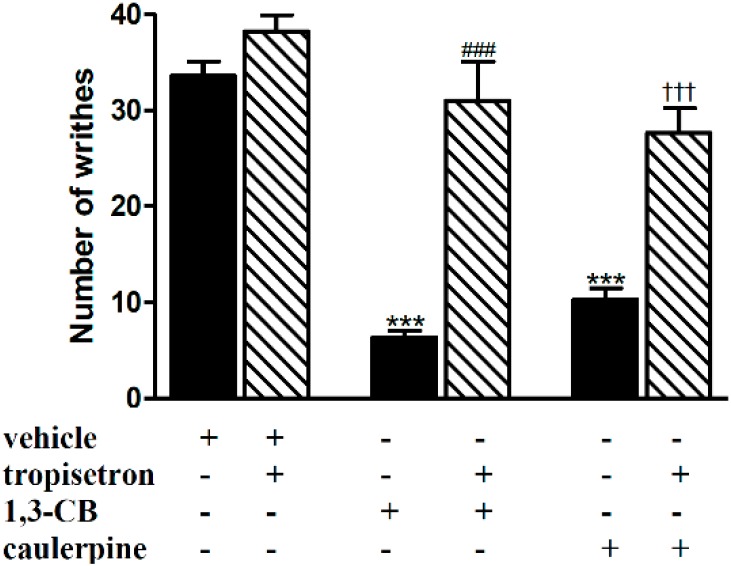
Effect of tropisetron on the antinociceptive effect of caulerpine in the acetic acid model of nociception. Black columns: treatments alone; hatched columns: pretreatment and treatments. The results are expressed as means ± S.E.M. of six animals. (One-way ANOVA followed by Bonferroni’s test). *** *p* < 0.001 compared to vehicle; ^###^ *p* < 0.001 compared to 1,3-CB; ^†††^ *p* < 0.001 compared to caulerpine.

The antinociception elicited by caulerpine seems to be dependent on 5-HT_3_ receptors since pretreatment with tropisetron, an antagonist of 5-HT_3_ receptors, prevented the antinociception induced by caulerpine, as also observed with 1,3-CB, a selective 5-HT_3_ agonist (Figure 6). In addition, our previous work showed that caulerpine antagonized serotonin-induced contractions in the guinea pig ileum [5]. 5-HT_3_ receptors are strongly involved in this smooth muscle model, which reinforces the hypothesis that caulerpine acts through serotonergic receptors to reduce nociception [27,28]. Moreover, other studies have also related the action of indole compounds to 5-HT receptors [29]. It is noteworthy that at the moment we cannot carry out receptor binding assay to evaluate the interaction between caulerpine and 5-HT_3_ receptor. However, this study could be done in the near future in association with other experiments.

## 3. Experimental Section

### 3.1. Animals

Swiss mice of both sexes, 6–8 weeks of age with an average weight of 25–30 g, were obtained from the Central Animal House of the Federal University of Alagoas (Maceió, Brazil) and were used throughout the experiments. They were housed in single-sex cages under a 12-h light/dark cycle at constant temperature (22 ± 2 °C) with free access to water and pellet food. Eight hours before each experiment, the animals received only water, to avoid food interference with test substance absorption. The experiments were performed after the approval of the protocol by the Ethics Committee-UFAL for animal handling (No. 23065.017275/2011-94).

### 3.2. Drugs

Naloxone, morphine, flumazenil and diazepam were purchased from Cristália, (Itapira, SP, Brazil). Acetic acid, yohimbine, clonidine, atropine, pilocarpine, tropisetron, 1-(3-chlorophenyl)biguanide (1,3-CB), l-arginine, Nω-nitro-l-arginine (l-NOARG) were obtained from Sigma-Aldrich (St. Louis, MO, USA). These drugs were dissolved and diluted in saline (0.9% NaCl). Caulerpine was dissolved in Tween^®^ 80 and diluted in carboxymethylcellulose from Sigma-Aldrich.

### 3.3. Isolation of Caulerpine

*Caulerpa sertularioides* and *C. mexicana* algae were collected from the coastal region of Cabo Branco, João Pessoa, Paraíba State, Brazil in March 2009. The specimens were identified by Dr. George Emmanuel Cavalcanti de Miranda. Voucher specimens of *C. sertularioides* (JPB 13983) and *C. mexicana* (JPB 13985) have been deposited in the Lauro Pires Xavier Herbarium at the Federal University of Paraíba (Universidade Federal da Paraíba), Brazil. The alga was extracted with MeOH at room temperature and the extract was partitioned between hexane, dichloromethane, ethyl acetate and methanol. The orange red pigment obtained by precipitation in the ethyl acetate phase was assigned the structure of 5,12-dihydrocycloocta[1,2-b;5,6-b']diindole-6,13-dicarboxylic acid dimethyl ester, named caulerpine or caulerpin, based on its NMR spectral data and chemical properties [5].

### 3.4. HPLC Analysis

Chromatographic analyses were performed using a Shimadzu Prominence LG2OAT HPLC system (Shimadzu, Columbia, MD, USA) equipped with a photodiode array detector (SPDM2O) and a reversed-phase column (Phenomenex Luna, 4.6 mm × 150 mm × 5 µm). The mobile phase consisted of a mixture water (A) and methanol (B) at a flow rate of 1 mL/min. A gradient elution was used with 70%–100% B, 0–20 min. Chromatograms were recorded at 320 nm. The caulerpine showed 99.4% purity.

### 3.5. Mechanism of the Antinociceptive Action of Caulerpine

To address some of the mechanisms through which caulerpine produces antinociception in the writhing test, animals were treated with different drugs. In this test, mice were injected i.p. with 0.1 mL/10 g of 0.6% acetic acid solution 40 min after the administration of caulerpine (40 mg/kg, p.o.), positive controls or vehicle. The writhings were counted for 20 min after a latency period of 5 min [30].

#### 3.5.1. Involvement of the Opioid, l-Arginine-Nitric Oxide, Adrenergic, Cholinergic, GABAergic and Serotonergic Pathways

To assess the possible participation of the opioid system in the antinociceptive effect of caulerpine, mice were pretreated with naloxone (1 mg/kg, i.p.) 20 min before receiving caulerpine (40 mg/kg, p.o.), morphine (5 mg/kg, i.p.) or vehicle (10 mL/kg, p.o.) [31]. Nociceptive responses to acetic acid were recorded 40 min after the administration of caulerpine, morphine or vehicle. As a control group, animals were pretreated with vehicle (10 mL/kg, i.p.) instead of naloxone. Such controls were used also in all the experiments described below. To assess the participation of other pathways in the antinociceptive effect of caulerpine, similar experiments were performed by changing only the drugs used for the pretreatment and as positive controls (Table 1).

**Table 1:** Drugs used to evaluate the mechanism of antinociceptive action of caulerpine.

Pathway	Pretreatment	Positive Control	Reference
Opiod	Naloxone (1 mg/kg, i.p.)	Morphine (5 mg/kg, i.p.)	[31]
Adrenergic (α_2_)	Yohimbine (0.15 mg/kg, i.p.)	Clonidine (0.1 mg/kg, i.p.)	[25]
Cholinergic (muscarinic)	Atropine (1 mg/kg, i.p.)	Pilocarpine (3 mg/kg, i.p.)	[32]
GABAergic (GABA_A_)	Flumazenil (2 mg/kg, i.p	Diazepam (1.5 mg/kg, i.p.)	[33]
Serotonergic (5-HT_3_)	Tropisetron (1 mg/kg, i.p.)	1-(3-chlorophenyl)biguanide (1 mg/kg, i.p.)	[25]
l-arginine-NO	l-arginine (40 mg/kg, i.p.)	l-NOARG (75 mg/kg, i.p.)	[25]

#### 3.5.2. α_2_-Adrenoceptor Binding Assay

Adult male Wistar rats (250–300 g) were anaesthetized with ether and killed by decapitation. The brains were rapidly removed on ice and cortex were dissected, weighted and stored in liquid nitrogen, according to a procedure approved by the Institutional Ethical Committee for Animal Care from the Federal University of Rio de Janeiro. The cortex were homogenized in a Potter apparatus with a motor-driven Teflon pestle at 4 °C in 10 volumes of ice-cold Tris-HCl 50 mM buffer (pH 7.4) per gram of tissue and then centrifuged twice at 900 g_max_ at 4 °C for 10 min. The resulting supernatants were combined and ultracentrifuged at 48,000 g_av_ for 10 min. The pellet was resuspended in buffer and incubated at 37 °C during 10 min for endogenous neurotransmitters removal. This suspension was cooled on ice and ultracentrifuged twice at 48,000 g_av_ for 10 min at 4 °C. The final pellet was resuspended in buffer (1.5 mL/g tissue) and stored in liquid nitrogen. As previously described [34], cortical membranes from rat brain (150 µg protein) and 1 nM [^3^H]-RX821002 (60 Ci/mmol, New England Nuclear Life Science Products, Perkin Elmer, Boston, MA, USA) were incubated at 30 °C for 45 min under yellow light in a solution containing 50 mM Tris-HCl (pH 7.4), in a final volume of 500 µL in the presence or absence of 3 µM caulerpine. Non-specific binding was estimated in the presence of 100 µM (‒)-epinephrine. After incubation, samples were rapidly diluted with 3 × 4 mL Tris-HCl 5 mM and immediately filtered under vacuum on glass fibre filters (GMF 3, Filtrak, Bärenstein, Germany) previously soaked in 0.5% polyethyleneimine. Filters were then dried and immersed in a scintillation mixture (1,4-bis-[2-(5-phenyloxazolyl)]-benzene—POPOP—0.1 g/L and 2,5-diphenyl-oxazole—POP—4.0 g/L in toluene). The radioactivity retained in the filters was counted with a Packard Tri-Carb 1600 TR liquid scintillation analyzer (PerkinElmer, Waltham, MA, USA).

### 3.6. Statistical Analysis

Data obtained from animal experiments were expressed as mean and standard error of the mean (mean ± S.E.M.). Statistical differences between the treated and control groups were evaluated by ANOVA followed by Bonferroni’s test. *p* < 0.05 was considered statistically significant.

## 4. Conclusions

In summary, the present study provides evidences that the antinociceptive effect of caulerpine is dependent on α_2_-adrenoceptors and 5-HT_3_ receptors. Thus, acting on these two pathways, caulerpine may be considered a lead compound for the development of new dual-action analgesic drugs.
